# Graphene Quantum Dot-Based Electrochemical Immunosensors for Biomedical Applications

**DOI:** 10.3390/ma13010096

**Published:** 2019-12-23

**Authors:** Bhargav D. Mansuriya, Zeynep Altintas

**Affiliations:** Technical University of Berlin, Straße des 17. Juni 124, 10623 Berlin, Germany; b.mansuriya@campus.tu-berlin.de

**Keywords:** graphene quantum dots (GQDs), nanomaterials, electrochemical immunosensors, cancer diagnosis, infectious diseases, cardiovascular disorders

## Abstract

In the area of biomedicine, research for designing electrochemical sensors has evolved over the past decade, since it is crucial to selectively quantify biomarkers or pathogens in clinical samples for the efficacious diagnosis and/or treatment of various diseases. To fulfil the demand of rapid, specific, economic, and easy detection of such biomolecules in ultralow amounts, numerous nanomaterials have been explored to effectively enhance the sensitivity, selectivity, and reproducibility of immunosensors. Graphene quantum dots (GQDs) have garnered tremendous attention in immunosensor development, owing to their special attributes such as large surface area, excellent biocompatibility, quantum confinement, edge effects, and abundant sites for chemical modification. Besides these distinct features, GQDs acquire peroxidase (POD)-mimicking electro-catalytic activity, and hence, they can replace horseradish peroxidase (HRP)-based systems to conduct facile, quick, and inexpensive label-free immunoassays. The chief motive of this review article is to summarize and focus on the recent advances in GQD-based electrochemical immunosensors for the early and rapid detection of cancer, cardiovascular disorders, and pathogenic diseases. Moreover, the underlying principles of electrochemical immunosensing techniques are also highlighted. These GQD immunosensors are ubiquitous in biomedical diagnosis and conducive for miniaturization, encouraging low-cost disease diagnostics in developing nations using point-of-care testing (POCT) and similar allusive techniques.

## 1. Introduction

Over the last several years, cancer and cardiovascular diseases (CVDs) have become two major causes of death for several age groups worldwide. At the same time, several pathogenic diseases like bacterial and viral infections, as well as diseases caused by toxins, are prevailing across the globe. Diagnosis of such diseases by detecting them at a very early stage has led to the development of preventive medicines rather than conventional medicines (i.e., treatment-based), which is now quite possible by virtue of biosensors. The very first biosensor introduced was an electrochemical glucose sensor by the pioneers Clark and Lyon in 1960s [1]. Since then, biosensors are highly demanded and employed in biomedical applications, particularly for disease monitoring, drug discovery, and detection of biomolecules (i.e., disease biomarkers, pollutants, toxins, and disease-causing microbes) in biological samples such as blood, urine, saliva, sweat, food, and environmental contents [2,3,4,5,6,7,8].

**Figure 1:**
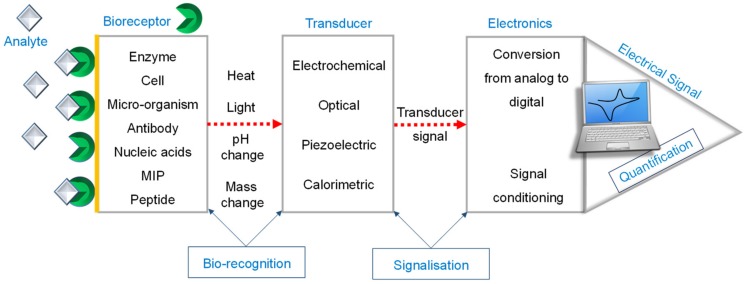
Schematic representation of a biosensor. MIP, molecularly imprinted polymer.

The International Union of Pure and Applied Chemistry (IUPAC) defines a biosensor as “a device that uses specific biochemical reactions mediated by isolated enzymes, immunosystems, tissues, organelles or whole cells to detect chemical compounds usually by electrical, thermal or optical signals” [9]. These analytical devices convert a biological or chemical response into an electrical signal and are usually classified on the basis of the type of bioreceptors involved in bio-recognition events, i.e., enzyme [10], antibody [11], peptide [12], aptamer [13], DNA [14], and molecularly imprinted polymer (MIP)-based sensors [15,16], or according to the type of transducer employed, such as electrochemical [17], optical [18], piezoelectric [19], and calorimetric biosensors [20]. The general working principle behind all of the biosensors is portrayed in Figure 1. Amidst these sensors, the one with antibody as a bio-recognition element is one of the most important and widely studied sensing platforms. Such platforms are often termed as immunosensor, since they function on the basis of immunoreaction (i.e., specific recognition) between antigens and antibodies [21,22]. They can meet the needs of specific and rapid identification of the bio-recognition molecules, as well as the on-line and real-time detection requirements of modern analytical procedures. Therefore, immunosensors can offer a broader platform for research and development.

Nanomaterials, the quintessential materials of nanotechnology with noticeably three-dimensional (3D) space, have sizes ranging within a nanoscale (1–100 nm) [23]. In the recent plot, the research regarding various nanomaterials is emerging with a giant footstep, due to which they are progressively becoming a sector of routine in terms of cosmetics, food safety, drug delivery, therapeutics, environmental sciences, biosensors, and many others [24,25,26]. By means of these, unprecedented paths for exposure of nanomaterials to living beings and the environment are expanding.

In the development of immunosensors, nanomaterials have been explored as electrode modifiers to enhance the antibody loadings due to their good adsorption ability, biocompatibility, and structural compatibility. Enduringly, these nanomaterials constitute discrete biological and physicochemical properties compared to their conventional counterparts, which bestow them favorable characteristics for the fabrication of biosensors. They exhibit surface effects, small size effects, and macroscopic quantum tunneling effects, hence they have unique mechanical, electrical, optical, magnetic, and catalytic properties as compared to the bulk materials [27,28,29,30]. Moreover, they have also been employed as nanocarriers for signaling elements (detector bioreceptors, enzymes, and/or electroactive label), as catalysts and electron transfer promoters for signal amplification [31,32,33,34], thus providing new approaches for the development and application of bioelectrochemical sensors.

To date, plenty of nanomaterials have been investigated as signaling species, including nanoparticles (NPs) [35,36,37], nanowires [38], carbon nanotubes (CNTs) [39], graphene [40,41,42], magnetic beads [43,44], and quantum dots (QDs) [45,46], to further improve the sensitivity, selectivity, and reproducibility of electrochemical immunosensors. Among these materials, QDs, like carbon dots and graphene quantum dots (GQDs), have gained popularity for their unique characteristics such as good biocompatibility, electro-catalytic activity, controllable size, good signal amplification, and multiplexed detection ability. They are chemically stable, water soluble, robust, inert, and photo-stable against blinking and photo-bleaching. Moreover, it is easy to synthesize and functionalize these nanomaterials [46,47,48,49,50,51,52].

**Figure 2:**
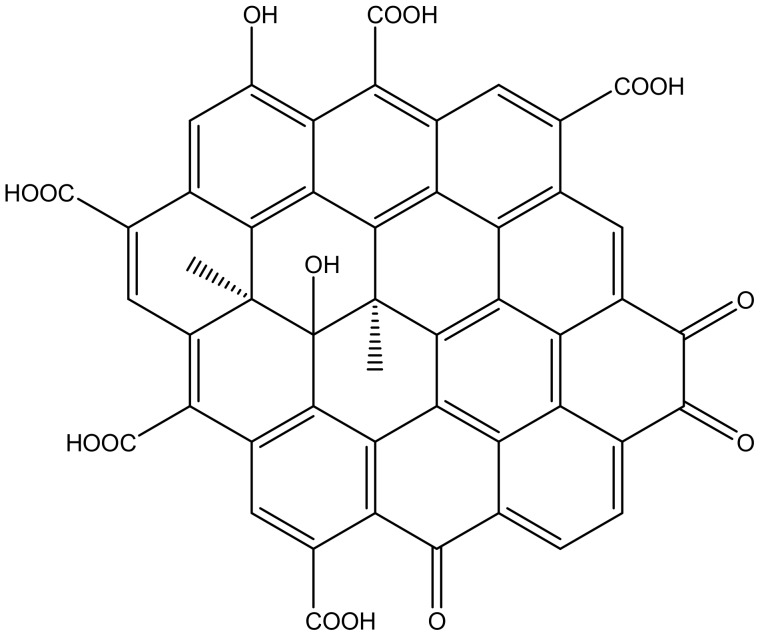
Structure of graphene quantum dots (GQDs).

GQDs are zero-dimensional (0D) carbon nanomaterials, composed of a framework analogous to graphene, with properties derived from both graphene and carbon dots [53]. They are extensively used for biological, optoelectronics, and environmental applications, and their phenomenal characterization has directed its pertinence in electrochemistry as well [46,54,55]. They are anisotropic with lateral dimension greater than the height, having mono- or multiple layers of graphene, and possess chemical groups on their edge that serves abundant sites for functionalization [46]. Figure 2 depicts the chemical structure of GQDs.

Being fluorescent nanoscale graphene fragments, GQDs result into exciton confinement in 3–20 nm particles and quantum-size effect [56,57]. Due to the fact that graphene is a zero band gap nanomaterial, it is non-luminescent, renders quantum confinement in fixed sized species, and has an infinite excitation Bohr radius [58]. However, GQDs contain a band gap because of size, edge effects, and quantum confinement that can be easily regulated by modifying their surface chemistry and size [55,58]. Unlike semiconductor QDs having two quantum states at a given energy level, GQDs exhibit four. These supplementary quantum states make them efficient for quantum computing [46,59]. Moreover, GQDs can be grafted with various other nanomaterials via π–π network to form hybrid nanomaterial [59].

Synthesis of GQDs with controllable size can be achieved by either top-down or bottom-up approaches [60]. In top-down methods, two-dimensional (2D) graphene or graphene oxide (GO) sheets, carbon fibers, CNTs, or graphite are dissected to form 0D GQDs, whereas in bottom-up processes, they are synthesized via stepwise reactions of small molecular precursors [54,57]. GQDs tend to form conjugates with proteins, nucleic acids, and antibodies, since they are identical to such compounds by virtue of their small size. They can enlarge the effective surface of immunosensors through the absorption of a considerable number of antibodies by providing large free room [48,61,62]. Besides, GQDs are capable enough to catalyze hydrogen peroxide (H_2_O_2_) by acting as nanozymes for the label-free detection of analytes [63]. They acquire peroxidase (POD)-mimicking catalytic properties, which lead to simultaneous oxidation and reduction of an electron–donor substrate and H_2_O_2_, respectively. In the field of biosensors, horseradish peroxidase (HRP), a peroxidase enzyme, is usually employed, where the labeling of a secondary receptor for target detection is a necessity, thus resulting in tedious and more expensive assay procedures [14]. To conduct these assays quickly and economically, GQDs can be employed to avoid HRP-labeled secondary antibodies [47,63,64].

Emerging research on GQDs in designing electrochemical immunosensors has been conducted incredibly in the last five years, owing to their attractive features [63,65,66,67]. Moreover, scientists have zeroed in on the development of such immunosensors for biomedical applications via the usage of (a) biomarkers or pathogens responsible for the respective disease type, (b) highly specific and sensitive immunosensors, and (c) various bioassays. According to the National Institutes of Health, a biological marker (biomarker) is defined as, “A characteristic that is objectively measured and evaluated as an indicator of normal biological processes, pathogenic processes, or pharmacologic responses to a therapeutic intervention” [68]. The use of biomarkers and pathogens in detecting and treating certain diseases in their very early phase is being assumed to develop continuously in coming years.

In this review, we discuss the working principle, new accomplishments, and progress of electrochemical immunosensors based on GQDs for several important biomedical applications, especially, for diagnosing and monitoring several types of cancers, cardiovascular diseases, and infections, before the direst of their symptoms take over. The research regarding the pivotal role of GQDs in designing and enhancing the performance of electrochemical antibody biosensors is a primary emphasis of this review, which summarizes the former studies of GQD-based electrochemical immunosensors and further expansion of their practical applications.

## 2. Electrochemical Sensors

Electrochemical sensors are supremely attractive when compared to optical and thermal sensors, owing to their unique detectability, experimental simplicity, and cost effectiveness. They have a prominent position among the currently accessible sensors that have reached the commercial stage and have been well-known for a wide range of important applications in the area of biomedicine [69,70,71]. This sensor type can function as a miniaturized device for point-of-care testing (POCT) [72,73].

**Figure 3:**
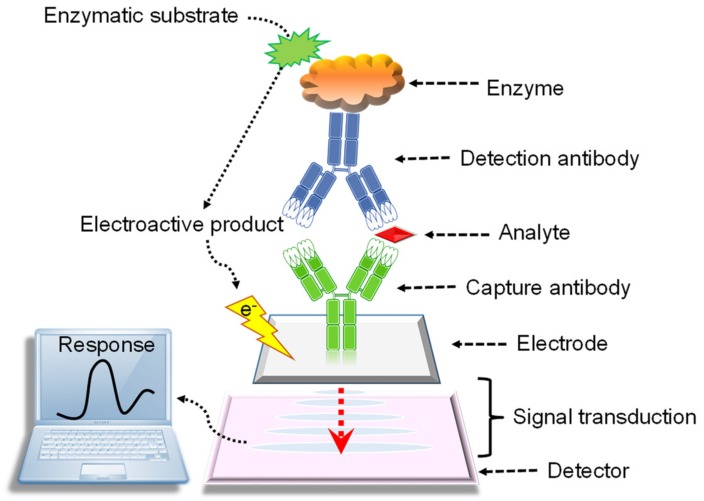
Analytical principle of electrochemical immunosensors.

Usually, electrochemical sensors comprise two basic elements, (a) a molecular recognition system which is the most significant part of a sensor, and (b) a physicochemical transducer system which is a component that converts the chemical or biological response into a signal that can be detected by modern electrical instrumentations. These two parts build a working (or sensing) electrode. A reference electrode, and often a counter electrode, are also engaged in the electrical measurements [74,75]. The IUPAC defined an electrochemical immunosensor as “an integrated device based on an antigen/antibody reaction, which can convert certain chemical substances or their concentration signals into a corresponding electric signal through the sensor element, and realize a specific quantitative or semi-quantitative analysis” [76]. As shown in Figure 3, these sensors are based on immunoassays, where the antibodies (by means of capture and detection agents) specifically bind to their respective antigens (analyte or target molecule) such as disease biomarkers, pathogens, toxins, or interact with components of the host’s immune system, i.e., the antibodies have high affinity towards their respective antigens [77,78].

**Figure 4:**
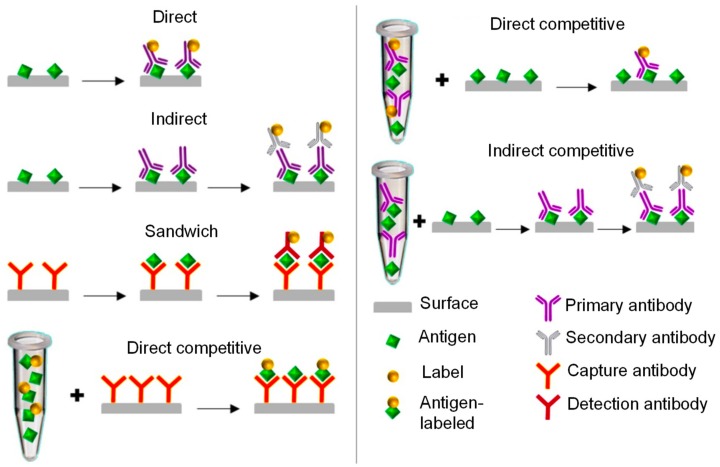
Various modes of bioassays employed in the development of electrochemical immunosensors [72].

In relation to immunoreactions, electrochemical antibody sensors can be based on various bioassay formats, for instance, direct, indirect, competitive, or sandwich modes. All of these immunoassays share a common basic principle (Figure 4) and generally involve the following steps [78]:

(a) Capture of the analyte of interest (usually target antigen);
(b) Occlusion of the non-reacted surface; and
(c) Recognition of the analyte.

Of all of the bioassay formats, the direct immunoassay is the most straightforward form of analyte detection. It covers the integration of a target molecule on the sensor surface, followed by washing as well as blocking steps, thenceforth allowing an effective immobilization of a specific labeled antibody for the recognition of a desired analyte. However, in an indirect immunoassay, a labeled secondary antibody is subjected to a specific primary antibody [72,79]. In the case of a sandwich assay, antigens are “sandwiched” between the capture and detection antibodies through two different binding sites [11,80].

The competitive assays are further classified into two: Direct and indirect forms. The former approach works either by competing free antigens with labeled antigens to interact with the immobilized antibodies, or by competing immobilized free antigens with each other to react with labeled primary antibodies, while the later approach involves the binding of a labeled secondary antibody to a primary antibody for determining the target analyte. The indirect competitive assay is usually preferred when the labeled primary antibodies are not available, which overcomes the issues of incorrect antibody immobilization and loss of affinity [71,72,78]. All of the immunoassays displayed in Figure 4 are based on the use of a label. They compute the signals generated by the label, leading to versatile and sensitive detection. Nevertheless, the immunosensors can also be label-free, which have the ability to encounter the physical changes during the immuno-complex formation [72,81,82].

Antibody biosensors can offer benefits of a wide linear response range, low detection limits, reproducibility, and good stability. Transduction of a biochemical reaction into an electrical signal can be accomplished either by amperometry, conductometry, impedimetry, potentiometry, or by voltammetry. This section entails the fundamentals of the often used electrochemical techniques for sensing biomarkers and/or pathogens. We elaborate a general outline of how the detection of different analytes can be achieved through such methods. Additionally, we describe their merits and limitations to guide the interested readers to choose the most suitable technique for a specific analysis.

### 2.1. Amperometric Sensors

**Figure 5:**
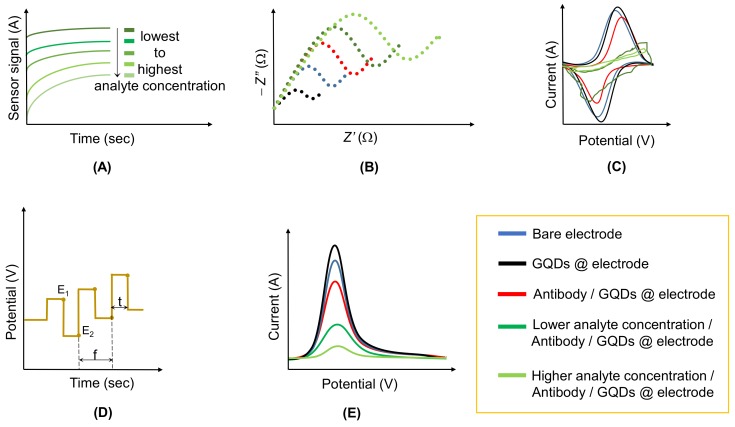
(**A**) Amperometric curves for the different concentrations of target analyte. (**B**) Nyquist plot: Change in the impedance upon electrode functionalization and analyte addition. (**C**) Cyclic voltammogram of a GQD-coated electrode, where its electrochemical response varies upon the specific binding of antibody to analyte. (**D**) Potential time profile of square wave voltammetry (SWV), where E_1_: Initial potential; E_2_: Potential after pulse; f: Pulse frequency; t: Pulse duration. (**E**) Representation of the analyte detection by means of GQD-coated electrodes, where the variation in current is proportional to the antibody occupancy.

Amperometric sensors measure a current flow generated by an electrochemical reaction at a constant voltage. The intrinsic principle behind these sensors is the specific molecular recognition of antigens by antibodies to establish a stable complex. Amperometric immunosensing can be (a) direct (non-labeled): Detection of the physical changes caused during immune complex formation; or (b) indirect (labeled): Using signal-generating labels. Since most of the protein analytes fail to act as redox couples, electrochemically-tagged labels are fused into the immunocomplex that results into the indirect measurement of the analyte. Besides, indirect amperometric immunosensing is usually preferred over the direct mode of measurement, owing to its high sensitivity and versatility [83]. These sensors involve the use of a potential applied between a working and a reference electrode to oxidize or reduce an electroactive species by measuring the resultant current [69]. Thus, current generated by the electrochemical reaction is directly proportional to the concentration of the electroactive species in the sample [84]. Figure 5A shows the ideal behavior of sensor signals generated by amperometric measurements.

### 2.2. Conductometric Sensors

Conductometric sensors measure the conductivity at a series of frequencies [69]. They are dependent upon conductance and a bio-recognition event. When a bio-recognition element interacts with an antigen, the current flow or conductivity of the solution is varied due to the change in concentration of ionic species [85]. Contrarily, there is a change in conductivity of the supporting electrolyte, when antibodies labeled with enzyme are fused to antigens in the sample solution, the enzymatic activity is inhibited by the antigen–antibody complex by blocking the surface of the electrode [17]. The resultant signal can be measured by an ohmmeter or multimeter. The conductometric detection obeys ohm’s law, i.e., S = χ × (A/L); where S is conductivity, χ is specific conductivity, A is area, and L is distance between the immersed electrodes. Advantages of sensors based on the conductometric principle include: (a) Propriety of thin-film electrodes for miniaturization, (b) reference electrode is not required, (c) transducers are not light-sensitive, (d) lower driving voltage to cut down the power consumption [86].

### 2.3. Impedimetric Sensors

A large number of research works have been emphasized on the perception of capacitive or impedimetric-based immunosensors. Impedance spectroscopy has been broadly employed for surface characterization, label-free detection, and determination of binding kinetics between biomolecules such as receptors, DNAs, proteins, antibodies, antigens, etc. [17,87]. The impedance is based on faradic or non-faradic measurements, i.e., in the presence or absence of a redox couple, respectively. The faradic immunosensors detect bio-recognition events taking place at the modified electrode by computing the change in the faradaic current, i.e., interfacial electron transfer resistance due to the steric hindrance caused by the biomolecular interaction and/or by the electrostatic repulsion between the free charges of the target molecules and the electroactive species in the supporting electrolyte [87,88].

Electron impedance spectroscopy (EIS) interprets the response of an electrochemical cell to a small amplitude sinusoidal voltage signal as a function of frequency. The resulting current sine wave alters in time (phase shift) with respect to the voltage wave, and this current–voltage ratio (*V(t)*/*I(t)*) gives the impedance (*Z*) [89,90]. To retrieve the information about the bio-reaction occurring at the interface, simulated circuit (i.e., Randles equivalent circuit) can be used to express charge transfer resistance (Rct), electrolyte resistance (Rel), Warburg impedance (W), mass transfer resistance (Rmt), and double-layer capacitance (Cdl) [91].

Bode and Nyquist plots are commonly used to interpret electrochemical impedance data. In the former plot, the total impedance (*|Z|*) is plotted against the frequency, while in the latter plot (Figure 5B), the imaginary part of impedance (*−Z″*) is plotted against the real part of impedance (*Z′*) [92]. In an electrochemical cell, diffusion phenomena, electrode kinetics, redox reactions, as well as molecular interactions on the surface of an electrode are akin to the resistors, capacitors, and inductors that impede the electrons’ flow in an alternating current (AC) circuit. Features that make these sensors attractive involve the ability to be miniaturized, remote control of implanted sensors, cost-effective electrode mass production, and economical instrumentation [89].

### 2.4. Potentiometric Sensors

The potential difference is measured by potentiometric immunosensors due to the immunocomplex formation between antibody and antigen [17]. These sensors are less sensitive, since the change in potential is small during the immunoaffinity reaction. Also, they are less accurate, less stable, and exhibit non-specific binding. Considering these limitations, potentiometric methods are less preferred over other electrochemical sensing techniques [93]. On the contrary, the ease of operation, use in automation, and miniaturization of solid-state sensors are the major advantages of such immunosensors [94].

### 2.5. Voltammetric Sensors

The general features of all voltammetric techniques are that they involve the application of a potential (*E*) to an electrode and control the resulting current (*i*) flowing through the electrochemical system. With time, the applied potential causes a change in the concentration of an electroactive species on the electrode surface via oxidation or reduction. The analytical merits of such techniques include excellent sensitivity with a wide concentration range for both organic and inorganic species, being able to work in a wide range of temperatures, rapid analysis, simultaneous detection of different analytes, and determination of kinetic parameters. Voltammetric sensing techniques include cyclic voltammetry, linear sweep/scan voltammetry, differential pulse voltammetry, square wave voltammetry, polarography, and stripping voltammetry. A two- or three-electrode electrochemical sensor containing a potentiostat can be employed to measure the current [95,96,97,98,99].

Cyclic voltammetry (CV) is based on varying the applied potential at a working electrode in both forward and reverse directions while controlling the current (Figure 5C). For instance, the initial scan could be in a negative direction to the switching potential, which would then be reversed and run in a positive direction. Depending on the analysis, one full cycle, a partial cycle, or a series of cycles can be performed [97].

Normal pulse voltammetry (NPV) amplifies a series of potential pulses in an increasing order. The current is measured near the end of each pulse. Usually, the duration of each pulse is 1–100 ms and the interval between each pulses is 0.1–5 s [95,96]. Differential pulse voltammetry (DPV) is relatable to NPV, where the potential is also scanned with a series of pulses. Nevertheless, it differs from NPV because each potential pulse is fixed, of small amplitude (10–100 mV). Current is measured twice for each pulse, i.e., just before the application of the pulse and at the end of the pulse [95,96].

The excitation signal in square wave voltammetry (SWV) comprises a symmetrical square wave pulse, where the forward pulse of the square wave coincides with the staircase step (Figure 5D). The peak height is directly proportional to the concentration of the electroactive species. SWV exhibits excellent sensitivity, ignores background currents, and enhances the signal to noise ratio [98,99]. Figure 5E depicts the SWV response with respect to the different steps tangled from the electrode modification to the analyte determination.

The main advantage of pulse techniques like DPV and NPV lies in the different decay rates of the faradaic and capacitive currents. The capacitive current is negligible as compared to the faradaic current, since it decays many folds faster than the faradaic current during each pulse. Such an increased ratio of the faradaic current to the capacitive current allows for a lower detection limit, which makes such methods suitable for the electrochemical detection of analytes [100]. Nevertheless, pulse strategies and SWV, are probably the most sensitive among all of the electrochemical characterization techniques, and hence most extensively employed for analytical purposes [101,102].

## 3. GQD-Based Electrochemical Immunosensors for Cancer Diagnosis

Cancer emerges from the transformation of normal cells into tumor cells in multiple stages, i.e., carcinogenesis/tumorigenesis or oncogenesis, that usually arises from a pre-cancerous lesion to a malignant tumor [103,104]. It has now become the leading cause of death in all age groups due to several factors, including exposure to certain radiations [105] and carcinogenic chemicals [106], infection by bio-carcinogens (e.g., certain bacteria, viruses, or parasites) [107], aging, genetic factors, geographic location, and improper and/or unhealthy diet [108,109].

Cancer rates and fatality are promptly sprouting across the globe. An estimation of around 18.1 million cancer cases and 9.6 million human deaths worldwide was reported in 2018 [110]. Considering the dramatic rise in cancer rates, recent advances in engineering electrochemical immunosensors have axiomatically improved the sensitivity required to detect very low concentrations of cancer biomarkers present in human biological fluids. Early stage biosensing of these analytes is the inaugural step towards hindering metastasis, adopting efficient therapy, and reducing mortality rate. Moreover, some immunosensors possessing multiplexing capability have also been reported for simultaneous detection of multiple cancer biomarkers [111,112]. Herein, we reviewed the recently developed GQD-based electrochemical immunosensors for cancer diagnosis. Important features of these sensors, such as the choice of electrode, assay type, electrochemical sensing technique, investigation range, and limit of detection, are also summarized in Table 1.

**Figure 6:**
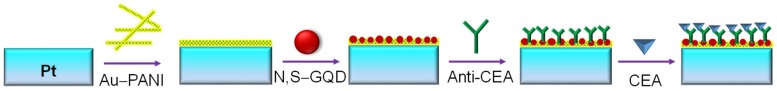
Stepwise construction of a label-free carcinoembryonic antigen (CEA) electrochemical impedimetric immunosensor.

Carcinoembryonic antigen (CEA) is one of the major tumor markers associated with the diagnosis and controlling of malignant tumors, such as pancreatic, colorectal, lung, liver, breast, and gastric cancers [113,114,115,116,117,118]. It is an oncofetal glycoprotein with a molecular weight of 180–200 kDa [119], which is generally expressed by mucosal cells and overexpressed by various malignancies [120,121]. Elevated level of CEA in human blood (>5 ng mL^−1^) is an indication of cancer cell formation [116]. Very recently, Ganganboina et al. developed a label-free impedimetric immunosensor based on nitrogen- and thiol-doped GQDs (N,S–GQDs) and gold-embedded polyaniline (Au–PANI) nanowires for the ultrasensitive and extremely selective detection of CEA [122]. The excellent electro-conductivity of N,S–GQDs/Au–PANI nanowires enhance the electron transfer. Figure 6 shows the immobilization of N,S–GQDs onto the Au–PANI surface via Au–thiol linkage after depositing Au–PANI onto the Pt electrode. N,S–GQDs act as the bifunctional probe to link anti-CEA and to amplify the electrochemical activity. The detection principle of CEA was based on the change in impedance of N,S–GQDs/Au–PANI after the introduction of CEA, suppressing the electron transfer after the conjugation of antibody–antigen on the N,S–GQDs/Au–PANI surface. This label-free immunosensor displays a wide linear range from 0.5 to 1000 ng mL^−1^, with a limit of detection (LOD) of 0.01 ng mL^−1^.

**Figure 7:**
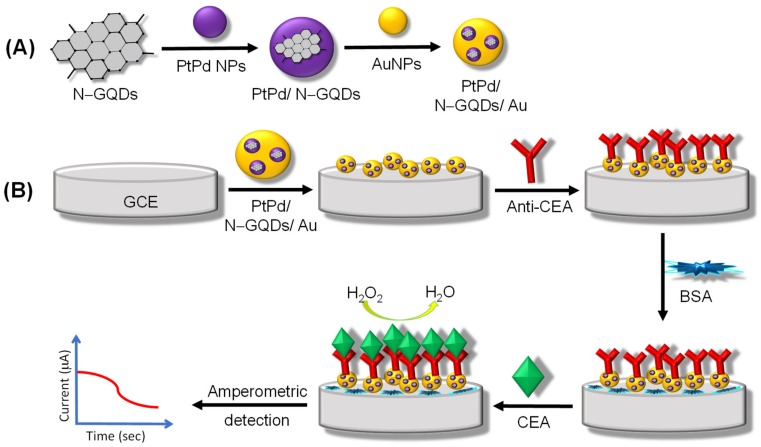
(**A**) Synthesis of PtPd/N–GQDs/Au nanocomposites. (**B**) Set-up of the label-free electrochemical amperometric immunosensor for CEA detection.

Another label-free immunosensor for the quantification of CEA based on PtPd/N–GQDs/Au functionalized glassy carbon electrode (GCE) was fabricated by Yang et al. in 2017 [67]. Preparation of PtPd/N–GQDs/Au via a self-assembly approach due to covalent binding is depicted in Figure 7. The synergistic effect of nanocomposites in PtPd/N–GQDs/Au provide the electro-catalytic activity towards hydrogen peroxide (H_2_O_2_) reduction, good biocompatibility, excellent conductivity, and large surface area; thus, PtPd/N–GQDs/Au was employed as transducer to effectively immobilize capture antibodies and to serve as a signal amplification platform. The specificity of this sensor was investigated against non-specific biomolecules like hepatitis B surface antigen (HBS), prostate specific antigen (PSA), human immunoglobulin (IgG), and BSA, whereas the variation in amperometric response of these samples with interference and CEA was found less than 5% of that without interferences, suggesting that the designed immunosensor is highly selective. This label-free amperometric immunosensor could attain high sensitivity and long-term stability for the detection of CEA, with a linear calibration plot ranging from 5 fg mL^−1^ to 50 ng mL^−1^, and the LOD was found as 2 fg mL^−1^.

An antibody sensor exploring the role of GQDs for quantitative determination of CEA was constructed by Nie and coworkers [123]. Here, the sensing strategy involved reinforcement of GQDs with poly(5-formylindole)/electrochemically-reduced graphene oxide nanocomposite (P5FIn/erGO) and Au nanoparticles (AuNPs). As an effective matrix, P5FIn/erGO nanocomposite facilitates the ion transport during the redox reactions and offers a large free-room for the bio-immobilization of primary antibody, whereas both GQDs and AuNPs as labels improve electron transfer efficacy during their conjugation with secondary antibody. By virtue of such multiple signal amplification properties of P5FIn/erGO and GQDs/AuNP, the as-prepared sandwich immunosensor led to quantify the target biomarker, allowing a dynamic linear range from 0.1 pg mL^−1^ to 10 ng mL^−1^ in human serum, with a detection limit of 3.78 fg mL^−1^.

Several studies have demonstrated that overexpression of interleukin-13 receptor α2 (IL13Rα2) can be found in a variety of human cancer cells such as colorectal, glioma, squamous cell carcinoma of head and neck, and AIDS-associated Kaposi’s sarcoma [130]. Very recently, Serafín and coworkers introduced an integrated amperometric electrochemical immunosensor for the determination of IL-13Rα2, which involves the immobilization of a biotinylated specific capture antibody onto streptavidin-modified screen-printed carbon electrodes (SPCEs) through grafting with p-amino benzoic acid (p-ABA) and further surface activation via EDC/NHS chemistry [124]. A hybrid nanomaterial comprising multi-walled carbon nanotubes (MWCNTs) and GQDs was opted as a nanocarrier of the detector antibody and HRP molecules. The use of this hybrid material considerably improves the assay due to the peroxidase-like activity of GQDs. Amperometric detection of IL-13Rα2 by H_2_O_2_/hydroquinone (HQ) system revealed a wide dynamic concentration range (2.7–100 ng mL^−1^), with an LOD value of 0.8 ng mL^−1^, and can be applied for quick and selective determination of IL-13Rα2 in raw cell lysates from human colorectal cancer cells.

In another study, the same group established a dual electrochemical immunoassay for the simultaneous detection of IL-13Rα2, as well as CDH-17, present in lysates from breast and colorectal cancer cells, respectively, with different metastatic potential [112]. Herein, MWCNT/GQD-functionalized screen-printed dual carbon electrode (SPdCE) was assembled to form a sandwich assay. The preparatory steps for integrating the electrode surface, as well as the detection strategy, were carried out similarly as reported in their aforementioned work. This dual amperometric sensor could selectively determine both biomarkers, i.e., IL-13sRα2 and CDH-17, with respective LOD values of 1.4 ng mL^−1^ and 0.03 ng mL^−1^.

**Figure 8:**
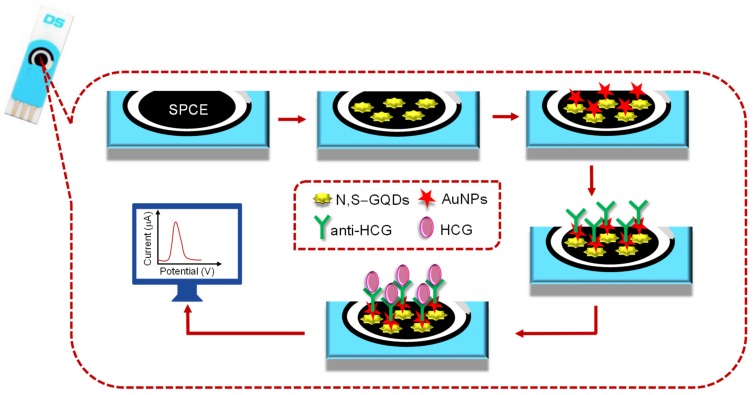
Experimental steps to fabricate an SPCE for monitoring HCG levels.

In 2019, Roushani and Valipour constructed an economic, facile, and label-free electrochemical immunosensor for the accurate and selective quantification of human chorionic gonadotropin (HCG) in human serum [61]. The N,S–GQDs, as well as AuNPs, were casted one after the other on SPCE, as illustrated in Figure 8. This modification not only increased the antibody loading, but also improved the electrochemical signal for protein analysis, thereby effectively enhancing the sensitivity of the sensor. In order to electrochemically characterize the surface modifications, the electrode was submitted to CV and EIS techniques, whereas DPV was used for the quantification of HCG by scanning the potential of −0.1 to 0.5 V. To study the reproducibility of the proposed sensor, the inter-assay, as well as intra-assay, precisions were performed for the same HCG levels with five measurements. As a result, the relative standard deviations (RSDs) were found to be 2.9% and 3.5%, respectively. This suggests that the N,S–GQD/AuNP-modified SPCE has a wide potential window with good reproducibility.

In 2018, an electrochemical immunosensor based on ternary signal amplification strategy to modify gold electrode was designed by Hasanzadeh et al. for the ultrasensitive recognition of p53, a tumor suppressor protein [125]. In this research, biotinylated p53 antibody was immobilized onto a green and biocompatible nanocomposite film consisting of poly L-cysteine (P-Cys) as polymetric conductive matrix and GQDs/AuNPs as synergetic amplification elements. A blend of such nanocomposites increases the effective surface area to immobilize a large number of anti-p53 antibodies. Under optimized conditions, the sensor provided a linear response between 0.0488 and 12.5 pM and an LOD of 23.4 fM.

**Figure 9:**
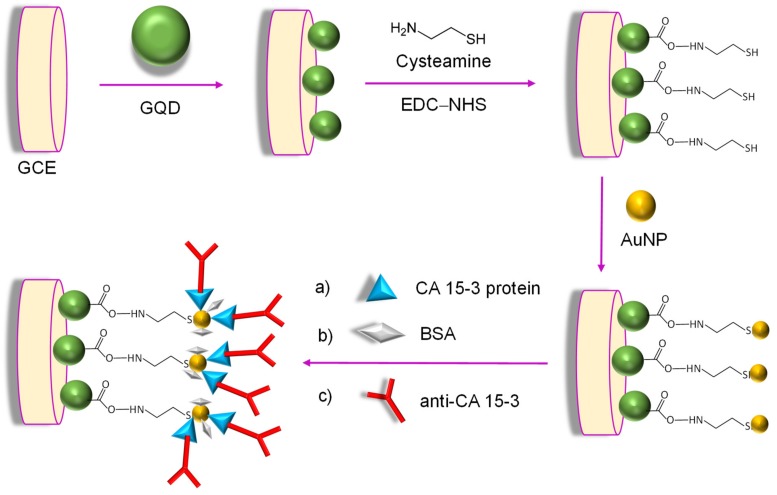
Schematic presentation of an electrochemical immunosensor for the detection of CA 15-3.

The same research team has also reported a label-free immunoassay for the accurate quantification of a breast cancer-specific protein, CA 15-3 [126]. In this approach, modification of GCE surface was initiated by electrochemically assembling the AuNPs onto thiolated graphene quantum dots using cysteamine (CysA), as well as 1-Ethyl-3-(3-dimethylaminopropyl)-carbodiimide (EDC) and N-hydroxysuccinimide (NHS). The subsequent CysA/AuNPs/GQDs hybrid interface provided stability and large loading of CA 15-3 molecules for their effective immobilization, thereby increasing the number of binding events occurring between the antigen and antibodies (Figure 9). The sensor fabrication, as well as the immunoreaction, were investigated by CV and SWV techniques, where the drop in the SWV peak current of [Fe(CN)_6_]^3−/4−^ was attributed to the response of the CA 15-3 antibody binding to the sensor. The prepared device can be applied to CA 15-3 malignant cell line lysates (human breast adenocarcinoma cell line-MCF-7) for breast cancer diagnosis.

Wu et al. proposed a label-free electrochemiluminescent immunosensor for prostate-specific antigen (PSA) using GQDs as GCE modifiers [127]. Incorporation of aminated as well as acarboxyl GQDs on gold/silver nanoparticle-reduced graphene oxide (Au/Ag–rGO) further increased the surface area of GCE and electron transferability, resulting in increased electrochemiluminescence (ECL). However, anti-PSA immobilization on the surface of modified electrode reduced the ECL intensity through the adsorption of Au/Ag toward proteins. The resulting immunosensor responded a calibration curve of PSA concentration in the range of 1 pg mL^−1^–10 ng mL^−1^, with an achievement of 0.29 pg mL^−1^ as the detection limit.

Considering PSA as a model analyte, a sandwich-type electrochemical sensor was also engineered for its quantification in human serum [128]. During the sensor preparation, GQD-functionalized graphene sheets (GS) were employed as labels to conjugate primary as well as secondary anti-PSA antibodies. Moreover, the authors reported electrochemical detection of PSA by SWV technique with a wide range of linear response (0.005–10 ng mL^−1^) and high sensitivity (LOD: 3 pg mL^−1^). This can be ascribed to the increased loadings of antibody on the electrode surface and good conductivity provided by such nanomaterials.

**Figure 10:**
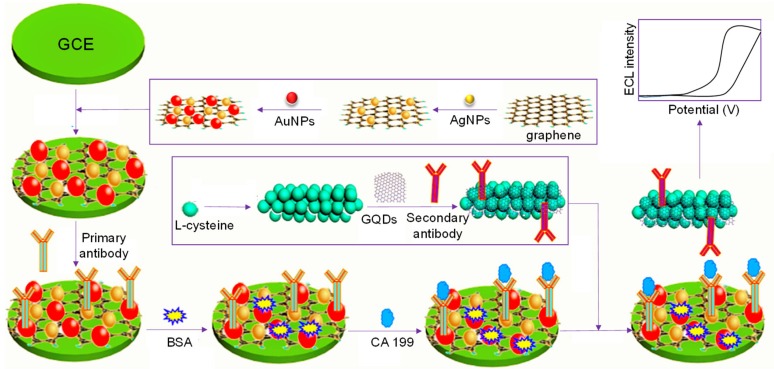
Casting procedure of a sandwich-based immunosensor for CA 199 detection [129].

Yang and coworkers assembled an ECL immunosensor by modifying a GCE surface for the selective determination of carbohydrate antigen 199 (CA 199), a potent tumor marker in diagnosing pancreatic cancer [129]. As indicated in Figure 10, the fabrication process involved the integration of a sensor surface using gold–silver nanocomposite-functionalized graphene (GN–Ag–Au) and porous PtPd nanochain-implanted GQDs (PtPd–GQDs). Owing to the favorable physical and chemical properties exhibited by these hybrid nanomaterials, GN–Ag–Au provided a large surface area to capture a huge number of primary antibodies and boosted the electronic transmission rate, while PtPd–GQDs delivered a large loading of secondary antibodies. This sandwich assay led to an improved sensitivity, achieving a broad detection range (0.002–70 U mL^−1^) and LOD of 0.96 mU mL^−1^ CA 199 concentration in human serum. Further, the authors reported that this biosensor was able to retain almost 96% of its initial activity, even after 7 weeks, and therefore has long-term stability, which could be attributed to the excellent biocompatibility of GQDs, as well as the strong bonding between secondary antibodies and PtPd–GQDs.

## 4. GQD-Based Electrochemical Immunosensors for Monitoring Cardiovascular Diseases

CVDs are the disorders of heart and blood vessels, which include coronary heart disease, cerebrovascular disease, rheumatic heart disease, and other conditions [78]. CVDs account for almost one-third of all deaths worldwide, which results in significant morbidity [131], and four-fifths of CVD deaths are due to heart attacks and strokes. Individuals at risk of CVD may have symptoms such as raised blood pressure, glucose, and lipids, as well as overweight and obesity, which can be readily measured in primary care facilities. Identifying those at highest risk of CVDs and ensuring they receive appropriate treatment can prevent premature deaths [132]. It is therefore noteworthy to diagnose patients with high risk of acute myocardial infarction at an early stage, which can reduce cost by screening the hospital admissions process and focusing resources on those that are specifically at high risk [78]. Thus, prognostic biomarkers are required to be measured with minimally invasive methodologies to improve the management of CVDs. These biomarkers, which are detected in the patient’s blood, can provide clinical evidence help in disease prognosis based on the change in level of certain cardiac biomarkers with respect to the severity of a particular CVD [133,134,135].

Electrochemical immunosensors hold divergent marvellous features, rendering them highly appropriate for the quantification of CVD biomarkers at very low concentrations in biological fluids. The exclusive benefits of these sensors in terms of high sensitivity and stability conveyed by nanostructuring the sensor surface, combined with high affinity and selectivity of bioreceptors, have resulted in the development of innovative electrochemical immunosensing strategies, which have been introduced as impressive substitutes to conventional techniques for clinical diagnosis and monitoring of CVD. To the best of our knowledge, only four GQD-based electrochemical immunosensors have been reported to date for detecting CVD biomarkers. A brief overview of these sensors is shown in Table 2. It is worth noting that all of these GQD sensors were reported after 2016, implying that the use of GQDs for the development of electrochemical immunosensors is evolving.

**Figure 11:**
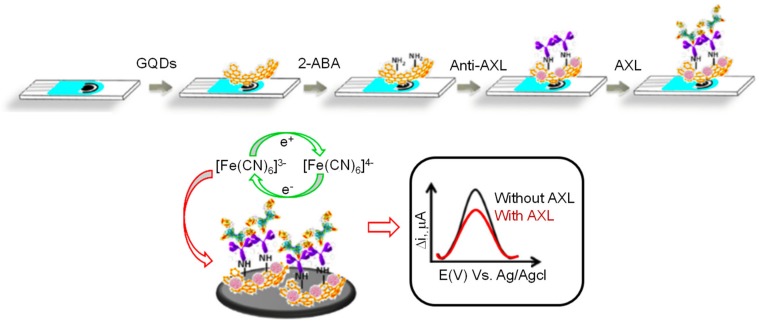
Display of the anti-AXL antibody immobilization on the GQDs/SPCE electrode through the oxidized sugar chains and electrochemical detection of AXL [136].

AXL is a tyrosine kinase receptor. The proteolytically refined extracellular portion of this protein (sAXL) is contemplated as a relevant biomarker in the pathophysiology of heart failure (HF). The level of sAXL in serum is elevated in HF patients, with a threshold value of 71 ng mL^−1^ [139,140]. Mollarasouli et al. recently engineered a label-free disposable electrochemical immunosensor for the detection of AXL, where functionalization of SPCE/GQDs involved the electropolymerization with 2-aminobenzyl amine (2-ABA) by cyclic voltammetry (20 cycles at 200 mVs^−1^ from 0.0 to −1.0 V vs. Ag pseudo-reference electrode) [136]. To ensure the adhesion of GQDs to the SPCE surface, the modified electrode was placed in an oven for 1 h at 120 °C. The specific anti-AXL antibody was subsequently immobilized through the stabilization of Schiff bases between amino-modified GQDs and aldehyde groups, induced on the antibody by periodate-mediated oxidation of their carbohydrate residues. After incubating the anti-AXL/GQDs/SPCE electrode for 1.5 h at room temperature, the immunorecognition of the target analyte was tracked by measuring the reduction in DPV response of the redox probe [Fe(CN)_6_]^3−/4−^, as represented in Figure 11. This modification led to an LOD of 0.5 pg mL^−1^.

Tuteja and co-workers fabricated a label-free impedimetric electrochemical immunosensor for the sensitive detection of cardiac myoglobin (cMyo) [66], an early indicator of acute myocardial infarction (AMI) [141]. Hydrothermally-synthesized GQDs were laminated on screen-printed electrodes (SPEs) as an immobilized template. Subsequent incubation of this electrode with the anti-myoglobin antibodies allowed the realization of a selective sensor system for myoglobin. The charge transfer resistance (R_ct_) values were generated as a function of varying antigen concentration and depicted a linear increase (from 0.20 to 0.31 kΩ) in the range of 0.01–100 ng mL^−1^ cMyo. This bioelectrode could also be regenerated for a minimum of five cycles. The estimated limit of detection, 0.01 ng mL^−1^, was almost 400 times improved when compared to the ELISA tests (estimated 4 ng mL^−1^).

**Figure 12:**
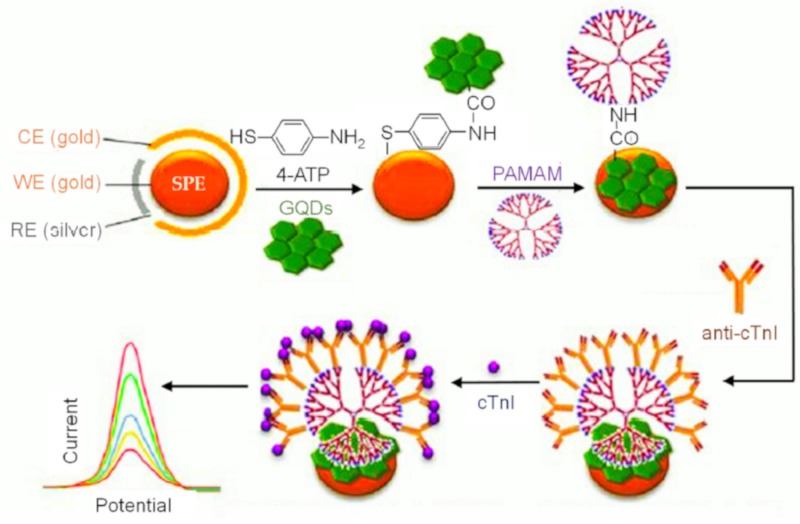
Various steps tangled in designing the label-free immunosensor for cTnI involving GQDs/PAMAM-modified SPGEs and immobilization of anti-cTnI. (CE: Counter electrode, WE: Working electrode, RE: Reference electrode). Adapted from [137].

In 2017, Bhatnagar et al. designed an ultrasensitive electrochemical immunosensor for the rapid sensing of cardiac troponin I (cTnI), a biomarker responsible for heart attack (myocardial infarction) in humans [137]. In this study, screen-printed gold electrode (SPGE) was embedded with a linker molecule 4-aminothiophenol (4-ATP) for amine termination of the electrode surface, and these amino groups were further coupled with carboxyl groups of GQD via EDC/NHS reaction. In order to enhance the sensitivity of the device, polyamidoamine (PAMAM) dendrimer was consecutively decorated on GQD through carbodiimide coupling. These hybrid nanomaterials act as gold electrode modifier to offer an ultra-high surface area for antibody immobilization. The activated cTnI monoclonal antibody was then mounted on PAMAM to form nanoprobe for sensing cTnI antigen (Figure 12). The recognition of cTnI was monitored by decrease in the [Fe(CN)_6_]^3−^ oxidation peak using cyclic voltammetry (CV) and differential pulse voltammetry (DPV), achieving a lower LOD of 20 fg mL^−1^ and a broad concentration range of cTnI (10^−6^–10 ng mL^−1^). Moreover, the as-prepared sensor was claimed to detect cTnI in human blood serum within 10 min.

Atrial fibrillation (AF) during open heart surgery can be interrupted by prophylactic beta-blockers (BB) [142]. In spite of carrying out plenty of studies during the past few years, adequate protection against AF in patients with post-coronary artery bypass graft surgery (CABG) has not been successfully achieved by BB. Assuming the relation between high C-reactive protein (CRP) levels and AF, it is debatable if high plasma CRP levels are only responsible for the occurrence of AF, or if raised CRP levels cause the disorder [143,144]. Bing et al. reported the use of GQDs as GCE modifiers to develop a proficient and effective electrochemical immunosensor for CRP detection in blood serum [138]. The stepwise construction of a receptive surface was characterized via EIS measurements. The R_ct_ values were target-specific, showing a linear relationship with logarithmic CRP concentration 0.5–70 nM and a low limit of detection of 176 pM. It is reported that this immunosensor could detect CRP in a single step and is able to detect clinical AF after CABG.

## 5. GQD-Based Electrochemical Immunosensors for the Detection of Infectious Diseases

Infections due to a plethora of microbes are a threat to the modern healthcare system. In total, there is a large number of about 1400 known species of disease-causing human pathogens, which mainly constitute water and foodborne micro-organisms like bacteria, virus, fungi, etc. [145]. Among these, bacterial and viral infections remain the predominant cause of mortality and morbidity, particularly in developing nations [146]. Lack of sanitation or poor access to treatment are the two major factors associated with such contagion. Besides, the prevalent use of pesticides is immensely and equally responsible for posing inimical health hazards and/or for polluting the environment with toxins.

The severity of the contagious diseases and widespread use of detrimental chemical residues have triggered breakthrough inventions in introducing versatile and portable biosensors for the rapid, specific, and sensitive detection of the target analytes in clinical as well as environmental sectors. However, numerous conventional culturing techniques are already in use for the identification and quantification of pathogens, but their limitations reside in less sensitivity, high cost instrumentation, requirement of highly skilled technicians and long assay times, and incapability to perform on-site monitoring. It is therefore a great challenge and quite significant to gain an on-field detection of microbes, as well as pesticide residues, in biological samples and to implement the preventive measures for their inactivation. This has now become considerably feasible thanks to the development of biosensors. In a broader range, electrochemical immunosensors have been extensively investigated by the use of multiplexing electrodes and nanomaterials like GQDs, where such sensor systems have been witnessed to conduct utterly inexpensive, reliable, ultrasensitive, and accurate quantification of antigens involved in environmental monitoring, as well as healthcare. Table 3 lists some major characteristics of GQD immunosensors for the detection of various bacteria, viruses, and toxins.

**Figure 13:**
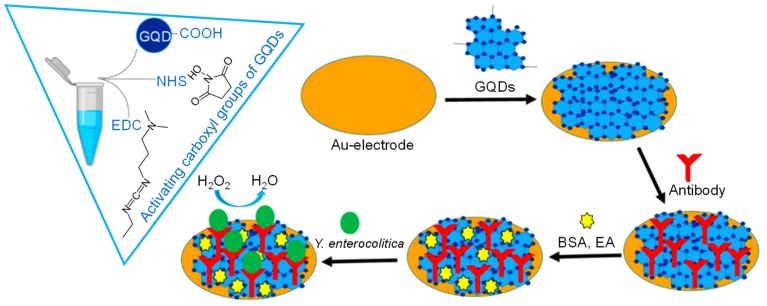
Steps involved in the development of a GQD-based immunosensor for *Y. enterocolitica* detection [63].

In 2019, Altintas et al. developed a novel, rapid, ultrasensitive, and highly specific label-free immunosensor approach for the efficacious diagnosis of *Yersinia enterecolitica*. The sensor preparation was initiated by enrichment of gold electrode surface with GQDs, owing to their enzyme-mimicking property to catalyze H_2_O_2_ [63]. In this approach, the bio-immobilization of antibodies was followed by inactivating unreacted carboxyl groups on the sensor surface with bovine serum albumin (BSA) and ethanolamine (EA), as depicted in Figure 13. The amperometric quantification of *Y. enterocolitica* at −0.2 V in the presence of H_2_O_2_ relied on the extent of inhibited electron transfer of the GQDs, which was blocked by the immunocomplex formation. As a result, signal reduction was observed with the increase in bacterial concentration. The resulted sensor exhibited a wide concentration range in complex media like milk and human serum, as mentioned in Table 3, with a very low detection limit of 5 cfu mL^−1^ and 30 cfu mL^−1^ in milk and human serum, respectively. It is worth mentioning that the matrix effect did not influence the sensor performance sufficiently, as the investigation range and the limit of detection in buffer were quite similar. The sensor revealed an LOD of 1 cfu mL^−1^ with higher electronic signals in buffer than those of complex matrices. Moreover, the specificity of the developed immunosensor was very high in the co-existence of several interfering bacteria (i.e., *Salmonella enteritidis, Bacillus anthracis, Escherichia coli and Yersinia pestis*), which further demonstrated the tempting characteristic of GQD-based immunoassays. This GQD sensor seems to be an attractive analytical tool that can pave the way for the identification of any pathogenic bacterium in clinical and food samples.

Tufa’s team constructed a sandwich immunosensor for the determination of *Mycobacterium tuberculosis* antigen (culture filtrate protein, CFP-10) containing a GQD-laminated Fe_3_O_4_@Ag core–shell nanostructure (Fe_3_O_4_@Ag/GQDs) and anti-CFP-10/AuNPs as GCE enhancer and labels for signal amplification, respectively [147]. This nanotriplex-based sensing platform rendered a noticeable synergetic electrochemical performance by the different functions of these nanomaterials, where Fe_3_O_4_ increased the surface-to-volume ratio; Ag improved electrical conductivity; and GQDs delivered large loading of the anti-CFP-10 antibody onto the electrode. Quantification of AuNPs by exposing the antigen–antibody complex to a potential of 1.3 V for 40 s and scanning by DPV revealed a wide linear range from 0.005 to 500 mg mL^−1^ with an LOD of 0.33 ng mL^−1^.

Depending on the ECL characteristics of nitrogen-doped graphene quantum dots (N–GQDs) and high selectivity of polydopamine (PDA) surface imprinted polymer (SIP), an articulately developed biosensor for the common food pathogen *Escherichia coli* O157:H7 was engineered by Chen at al. [148]. For the sensor preparation, dopamine and the target bacteria underwent direct electropolymerization on the electrode. CV (−0.5 to 0.5 V, 30 cycles) was performed with a scan rate 0.02 V s^−1^ to form PDA SIP. The established PDA SIP was subsequently immersed in acetic acid/SDS solution for 18 h for the removal of bacteria template to recognize *E. coli*. Accordingly, specific polyclonal antibody (pAb) was labeled with N–GQDs. The electrochemical properties of the transformed electrode were studied by collecting the EIS and CV stepwise. This sandwich assay could reliably detect concentrations of *E. coli* O157:H7 from 10 to 10^7^ cfu mL^−1^, with a detection limit of 8 cfu mL^−1^ in water samples.

**Figure 14:**
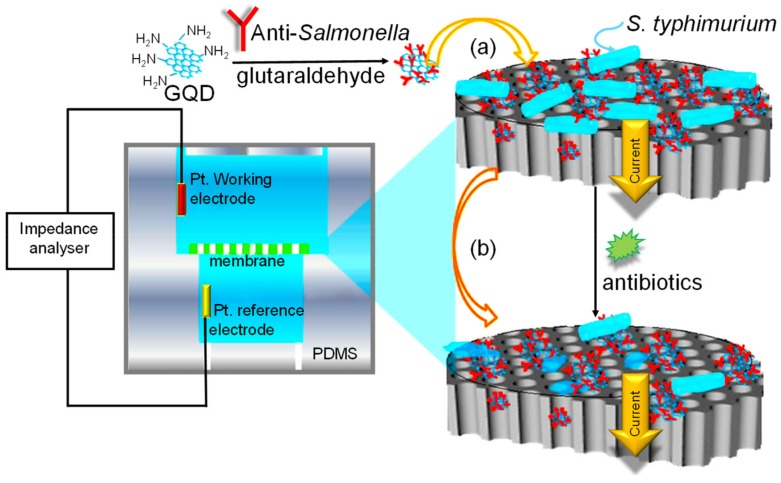
Impedimetric detection of *S. typhimurium* and its response to antibiotics: (**a**) Impedance increases; (**b**) impedance decreases. Adapted from [149].

Figure 14 embodies an impedimetric electrochemical biosensor consisting of a polydimethylsiloxane (PDMS) chamber integrated with a biofunctionalized nanoporous alumina membrane, where the sensing principle is based on the change in electrical impedance across the membrane, before and after the bacteria capture. The impedance increases due to the blockage of nanoporous skeleton by bacteria, and it decreases upon the addition of antibiotics because of bacterial cell deformation. In 2017, this capture/sensing mechanism was utilized and advanced by Ye et al. to rapidly determine the target bacteria, and furthermore to infer bacterial response to antibiotics [149]. In the study, *Salmonella typhimurium* was selected as a model pathogenic bacteria. The authors reported the use of amino-modified GQDs for altering the membrane to increase the surface-to-volume ratio. Ensuing, the GQD-rich alumina membrane was conjugated with the anti-Salmonella antibody by glutaraldehyde as a linker. Such an intriguing concept led to a detection limit of 1 pM, and exhibited a specificity for the *S. typhimurium* with a minimum cross-reactivity of about 5% to non-target bacteria. The proposed sensor can detect the target bacteria within 30 min, and hence, it has conceivable clinical application for the diagnosis of several other bacterial infections.

In 2017, Valipour and Roushani investigated the use of silver nanoparticles (AgNPs)/thiolated graphene quantum dots (GQD–SH) as GCE-modifying nanocomposites, and riboflavin as redox probe for the label-free quantification of hepatitis C virus core antigen (HCV) [150]. AgNPs were immobilized on –SH groups of GQDs via bonding formation of Ag–S, and, consequently, the anti-HCV molecules were loaded on the surface by chemisorption between AgNPs and –NH2 groups of antibody. The specific recognition between antibodies and antigens was analyzed by computing the decrease in the oxidation signal reduction of riboflavin using DPV. The proposed immunosensing platform demonstrated a wide linear range (0.05 pg mL^−1^ to 60 ng mL^−1^), with a limit of detection of 3 fg mL^−1^, and was applied for the analysis of spiked human serum.

Very recently, Chowdhury et al. introduced a pulse-induced impedimetric immunosensor for hepatitis E virus (HEV) detection [151]. The working electrode (GCE) was assembled with N,S–GQDs and Au–PANI nanowires via an interfacial polymerization and self-assembly approach. The N,S–GQDs/Au–PANI nanocomposite was conjugated covalently with antibody and an external electrical pulse was introduced during the HEV accumulation step to enhance the sensitivity towards virus owing to the surface expansion of the virus particle, as well as the anti-HEV-conjugated polyaniline chain length. The as-prepared biosensor demonstrated its potentiality to detect discrete HEV genotypes collected from human serum and from fecal specimen samples of HEV-infected monkey with a broad concentration, as listed in Table 3. Besides, it was also reported that this sensor could exhibit similar sensitivity to that determined by real-time quantitative reverse transcription polymerase chain (RT-qPCR).

**Figure 15:**
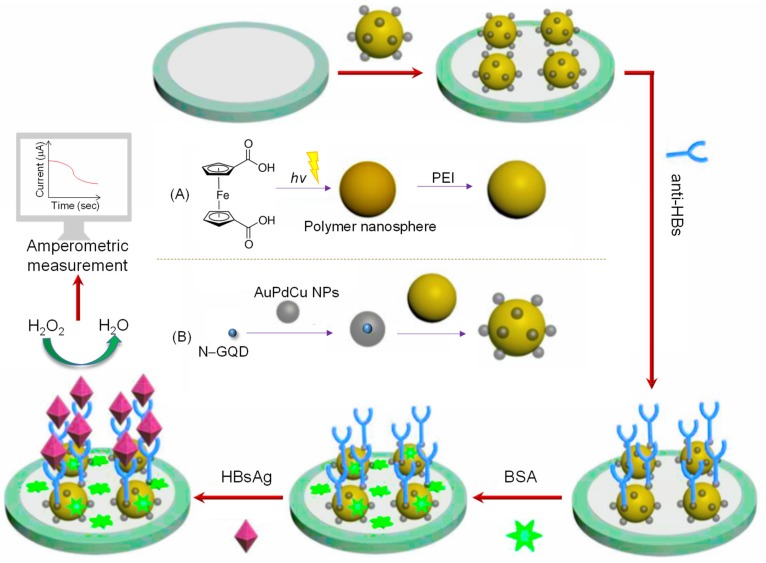
Schematic representation of a label-free impedimetric electrochemical immunosensor for the recognition of HBV antigen and the preparation procedure of: (**A**) Polymer nanosphere (PS) functionalized with polyethylenimine (PEI); (**B**) AuPdCu/N–GQDs@PS. Adapted from [82].

In 2018, another label-free GCE-based immunosensor was designed using a signal amplification system for the detection of hepatitis B surface antigen (HBsAg) [82]. In this work, N–GQDs supported surfactant-free AuPdCu ternary nanoparticles (AuPdCu/N–GQDs) delivered good electroconductivity and excellent catalytic activity for the reduction of H_2_O_2_. In addition to these nanomaterials, the electroactive polymer nanosphere (PS) integrated with polyethylenimine (PEI) was employed as an electronic mediator to load AuPdCu/N–GQDs and as a carrier to capture anti-HBs (Figure 15). Impedance measurements characterized the construction process of GCE, whilst amperometry determined the linear relationship between the current signal and HBsAg concentration (10 fg mL^−1^–50 ng mL^−1^) by scanning the potential at −0.4 V and achieved high sensitivity (LOD: 3 fg mL^−1^).

Ahmed et al. designed an immunosensing strategy based on GQDs and template-free in situ gold nanobundles (AuNBs) for the identification of fowl adenoviruses (FAdVs) [152]. The approach involved a modified layer-by-layer (LbL) technique to integrate AuNB film on carbon electrodes using L(+) ascorbic acid, gold chroloauric acid, and poly-l-lysine (PLL). The nanohybrid structure of AuNBs and GQDs were conjugated with specific anti-FAdVs antibodies prior to the quantification of antigen. Upon addition of the FAdV pathogen, a local electric signal enhancement revealed the detection of very low concentrations of target virus (up to 10 pfu mL^−1^) with 8.75 pfu mL^−1^ being the LOD. The proposed immunoassay yielded higher sensitivity (>100-fold) than the conventional ELISA.

Wang et al. reported an eminently sensitive and selective detection of avian leukosis virus subgroup J (ALVs-J) using Fe_3_O_4_-compounded GQDs and apoferritin-encapsulated Cu (Cu–apoferritin) nanoparticles for significantly enhancing the signal amplification [153]. In the proposed immunoassay, GQDs were employed for the immobilization of both primary as well as secondary ALVs-J antibodies, and at the same time, Cu–apoferritin nanoparticles were preferred as electroactive probes to be immobilized onto Fe_3_O_4_@GQDs hybrid. Before electrochemical detection of the target virus, the thoroughly established sandwich-type assembly was immersed in an HCl solution (pH = 2) for 20 min to liberate Cu from apoferritin cavity that led to the quantification of ALVs-J virus, ranging from 102.08 to 104.50 TCID_50_ mL^−1^, with a detection limit of 115 TCID_50_ mL^−1^.

**Figure 16:**
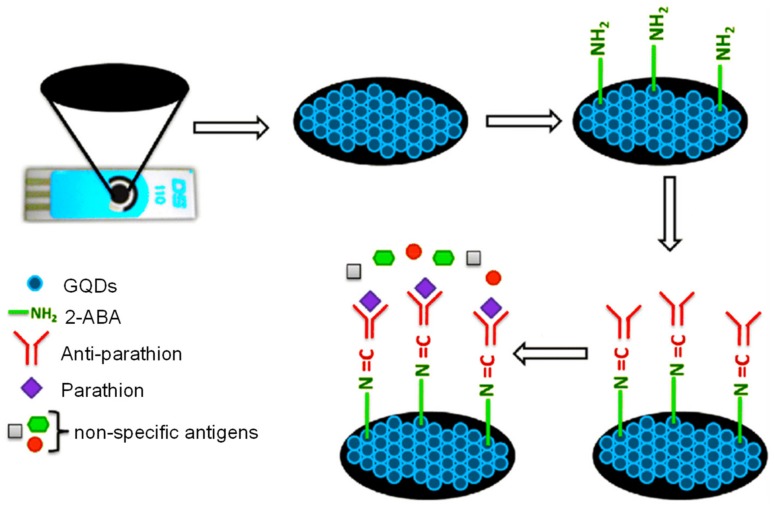
Immunosensing of parathion by GQD-functionalized SPCE [154].

Mehta et al. reported a functionalized-GQD-based label-free impedimetric antibody sensor for a pesticide, parathion [154]. To prepare the sensor, GQDs were laminated on an SPCE as an electrochemical substrate, and subsequently modified with 2-ABA to impart –NH_2_ functionality. The electrode was then conjugated with the specific anti-parathion antibodies (Figure 16). This electrochemical sensor attained a dynamic linear response for parathion within the range of 0.01–10^6^ ng L^−1^, with a very low detection limit of 46 pg L^−1^. According to the analysis of potential interferences, the as-developed strategy can specifically sense parathion in environmental and food contents, even in the presence of its metabolite, paraoxon. Moreover, the authors declared that this sensing platform can be reused by regenerating it with 10 mM glycine–HCl solution at least for five regeneration cycles, offering an economic detection principle for parathion.

**Figure 17:**
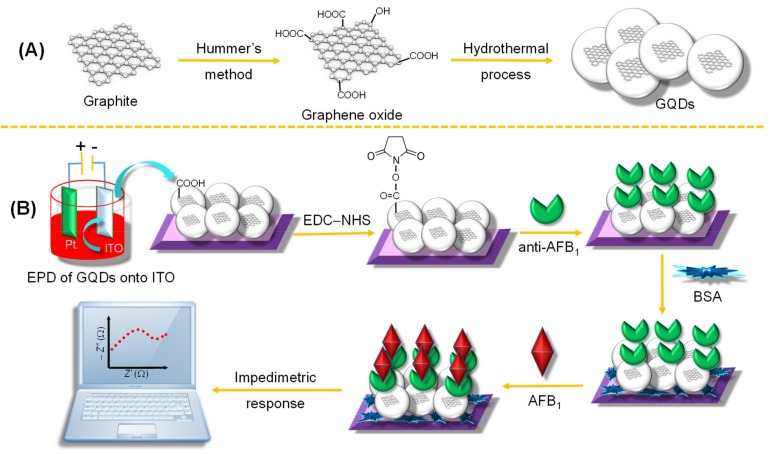
(**A**) Synthesis of GQDs. (**B**) EPD of GQDs onto indium tin oxide (ITO)-coated glass substrate and subsequent immobilization of anti-AFB_1_ for AFB_1_ identification.

Another label-free electrochemical sensing platform utilizing GQDs was developed in 2017 for the detection of a food toxin, aflatoxin B_1_ (AFB_1_) [155]. As shown in Figure 17, GQDs were chemically synthesized by slightly modifying the Hummer’s method [156]. The sensor set-up involved the casting of indium tin oxide (ITO)-coated glass electrode with hydrothermally synthesized GQDs through the electrophoretic deposition method (EPD), followed by a covalent amide bonding linkage (CO–NH) of monoclonal AFB_1_-antibodies using EDC–NHS as a cross-linker. The electrical characterization and biosensing studies were conducted by EIS and CV techniques. The large surface area, ease of electron transfer capability, and good electro-conductivity of the GQD-based immunosensor provided an improved detection limit (0.03 ng mL^−1^) and a wide detection range (0.1–2.0 ng mL^−1^) in aflatoxin B_1_-contaminated maize samples. Further, this biosensor was claimed to retain its activity up until 7 weeks with good reproducibility and storage stability.

## 6. Summary and Future Prospects

The quantification of disease biomarkers and pathogens in biological samples offer relevant information regarding the severity of certain diseases, even though their sensitivity and reliability are still a huge challenge due to their ultralow amount. However, the electrochemical immunosensors have now achieved the limit of detecting such analytes. The development of such sensors for early disease diagnosis is of vital significance for clinical therapy, disease monitoring, treatment, and discovery of preventive strategies. In this review, we discussed the outstanding contribution of GQDs in establishing innovative immunosensors by virtue of some of their fascinating attributes, such as excellent electro-catalytic activity, size and edge effects, numerous sites for functionalization, good electron transferability, signal amplification, photo-stability, etc. Hence, GQDs, individually or when combined with other nanomaterials, play a substantial role in functionalization of bio-electrodes, allowing quite an inexpensive, rapid, facile, stable, and reproducible immunosensing platform for practical applications. Additionally, such GQD-based electrochemical immunosensors are found to be highly specific, extremely sensitive, and very selective, even in complex media like milk, urine, human serum, etc., suggesting that their discriminative nature could be attributed to the physicochemical characteristics of GQDs.

On the contrary, the use of highly expensive nanomaterials with GQDs, tedious sensor assembly procedures, inadequate storage stability, and certain undesired aspects at nanoscale are some constraints that forbid their mass production. Plus, many of the recently reported GQD immunosensors have not been applied to the real world. Thus, approaches for manufacturing sensor batches and scaling-up to bulk production, as well as their validation in clinical samples, are still under the developing phase.

According to our detailed literature survey, 87% of GQD-based electrochemical immunosensors in biomedical diagnosis have been reported since 2016, highlighting the evolving characteristic of such sensing platforms. The use of GQDs for the fabrication of electrochemical immunosensors, along with the involvement of new biomarkers and pathogens, is being speculated to progress continuously and more significantly in the upcoming years. Further advances are expected to successfully achieve the development of GQD immunosensors for routine clinical applications.

## Figures and Tables

**Table 1:** Various GQD-loaded immunosensing platforms reported (2016 onwards) for cancer diagnosis.

Electrode	Nanomaterials	Biomarker	Assay Type	Technique(s)	Sample(s)	Linear Range	LOD	Reference
Pt-electrode	N,S–GQDs/Au–PANI	CEA	Direct	EIS	Human serum	0.5–1000 ng mL^−1^	0.01 ng mL^−1^	[122]
GCE	PtPd/N–GQDs/Au	CEA	Direct	Amperometry	Human serum	5 fg mL^−1^–50 ng mL^−1^	2 fg mL^−1^	[67]
GCE	P5FIn/erGO/GQDs/Au	CEA	Sandwich	ECL	Human serum	0.1–10 ng mL^−1^	3.78 fg mL^−1^	[123]
SPCE	MWCNTs/GQDs	IL-13Rα2	Sandwich	Amperometry	Raw cellular lysates from human CRC	2.7–100 ng mL^−1^	0.8 ng mL^−1^	[124]
SPdCE	MWCNTs/GQDs	IL-13Rα2, CDH-17	Sandwich	Amperometry	Raw cellular lysates from human CRC and breast cancer	4.92–100 ng mL^−1^ (IL-13sRα2) 0.11–10 ng mL^−1^ (CDH-17)	1.44 ng mL^−1^ (IL-13sRα2) 0.03 ng mL^−1^ (CDH-17)	[112]
SPCE	N,S–GQDs/AuNPs	HCG	Direct	CV, SWV, EIS	Human serum	0.1–125 pg mL^−1^	12.5 fg mL^−1^	[61]
Au-electrode	P-Cys/GQDs/AuNPs	p53	Direct	SWV, DPV	Human plasma	0.0488–12.5 pM	23.4 fM	[125]
GCE	CysA/AuNPs/GQDs	CA 15-3	Direct	SWV, CV	Human plasma; cellular lysates from human breast cancer	0.16–125 U mL^−1^	0.11 U mL^−1^	[126]
GCE	Au/Ag–rGO/GQDs	PSA	Direct	EIS, ECL	Human serum	1 pg–10 ng mL^−1^	0.29 pg mL^−1^	[127]
GCE	GQD/GS	PSA	Sandwich	SWV	Human serum	0.005–10 ng mL^−1^	3 pg mL^−1^	[128]
GCE	GN–Ag–Au/GQDs	CA 199	Sandwich	ECL	Human serum	0.002–70 U mL^−1^	0.96 mU mL^−1^	[129]

Abbreviations: Ag: Silver; AuNPs: Gold nanoparticles; CA 15-3: Carcinoma antigen 15-3; CA 199: Carbohydrate antigen 199; CEA: Carcinoembryogenic antigen; CDH-17: Cadherin-17; CRC: Colorectal cancer; CV: Cyclic voltammetry; CysA: Cysteamine; DPV: Differential pulse voltammetry; ECL: Electrochemiluminescence; EIS: Electron impedance spectroscopy; erGO: Electrochemically reduced graphene oxide; GCE: Glassy carbon electrode; GN: Graphene; GS: Graphene sheets; GQDs: Graphene quantum dots; HCG: Human chorionic gonadotropin; IL-13Rα2: Interleukin-13 receptor α2; MWCNTs: Multi-walled carbon nanotubes; N,S–GQDs: Nitrogen- and thiol-doped graphene quantum dots; p53: Tumor protein-53; P5FIn: Poly(5-formylindole); PANI: Polyaniline; P-Cys: Poly L-cysteine; Pd: Palladium; PSA: Prostate-specific antigen; Pt: Platinum; r-GO: Reduced graphene oxide; SPCE: Screen-printed carbon electrode; SPdCE: Screen-printed dual carbon electrode; SWV: Square wave voltammetry.

**Table 2:** GQD-linked immunosensors designed over the last five years to detect cardiac biomarkers.

Electrode	Nanomaterials	Biomarker	Technique(s)	Linear Range	LOD	Reference
SPCE	GQDs implanted with 2-ABA	AXL	DPV	1.7–1000 pg mL^−1^	0.5 pg mL^−1^	[136]
SPGE	PAMAM/GQDs	cTnI	CV, DPV	10^−6^–10 ng mL^−1^	20 fg mL^−1^	[137]
SPCE	GQDs	cMyo	CV, DPV, EIS	0.01–100 ng mL^−1^	0.01 ng mL^−1^	[66]
SPCE	GQDs	CRP	EIS	0.5–70 nM	176 pM	[138]

Note: All of the four abovementioned immunosenors follow direct mode of bioaassay. Abbreviations: 2-ABA: 2-aminobenzyl amine; AXL: Tyrosine kinase receptor; cMyo: Cardiac myoglobin; CRP: C-reactive protein; cTnI: Cardiac troponin I; CV: Cyclic voltammetry; DPV: Differential pulse voltammetry; EIS: Electron impedance spectroscopy; GQDs: Graphene quantum dots; PAMAM: Polyamidoamine; SPCE: Screen-printed carbon electrode; SPGE: Screen-printed gold electrode.

**Table 3:** Electrochemical antibody sensors involving the use of GQDs for pathogen detection.

Electrode	Nanomaterials	Pathogen	Assay Mode	Technique(s)	Sample(s)	Linear Range	LOD	Reference
Gold	GQDs	*Y. enterocolitica* (bacteria)	Direct	Amperometry	Milk and human serum	1–6.23 × 10^8^ cfu mL^−1^ (milk); 1–6.23 × 10^8^ cfu mL^−1^ (serum)	5 cfu mL^−1^ (milk); 30 cfu mL^−1^ (serum)	[63]
GCE	Fe_3_O_4_@AG/GQDs	CFP-10 (bacteria)	Sandwich	DPV	Human urine	0.005–500 μg mL^−1^	00.33 ng mL^−1^	[147]
GCE	PDA/N–GQDs	*E. coli* (bacteria)	Sandwich	ECL; CV; EIS	Water	10–10^7^ cfu mL^−1^	8 cfu mL^−1^	[148]
Platinum	GQDs	*S. typhimurium* (bacteria)	Direct	EIS	Buffer	1 pM–100 nM	1 pM	[149]
GCE	AgNPs/thiol–GQDs	HCV (virus)	Direct	DPV	Human serum	0.05 pg–60 ng mL^−1^	3 fg mL^−1^	[150]
GCE	N,S–GQDs/AuNPs/PANI	HEV (virus)	Direct	CV; EIS	Buffer, human serum, and feces of HEV-infected monkey	1–10^5^ fg mL^−1^ (feces of HEV-infected monkey); 10^2^–10^7^ RNA copies mL^−1^ (human serum)	0.8 fg mL^−1^ (feces of HEV-infected monkey); 96.7 RNA copies mL^−1^ (human serum)	[151]
GCE	AuPd/N–GQDs@PS	HBsAg (virus)	Direct	Amperometry	Human serum	10 fg mL^−1^–50 ng mL^−1^	3.3 fg mL^−1^	[82]
Carbon	GQDs/AuNBs	FAdVs (virus)	Sandwich	CV	Chicken blood	10–50 pfu mL^−1^	8.75 pfu mL^−1^	[152]
GCE	Fe_3_O_4_/GQDs/Cu-apoferritin	ALVs-J (virus)	Sandwich	DPV	Human serum	102.08–104.5 TCID_50_ mL^−1^	115 TCID_50_ mL^−1^	[153]
SPCE	GQDs/2-ABA	Parathion (toxin)	Direct	EIS	Food, water, and soil	0.01–10^6^ ng L^−1^	46 pg L^−1^	[154]
Glass	GQDs/ITO	AFB_1_ (toxin)	Direct	CV; EIS	Contaminated maize	0.1–2.0 ng mL^−1^	0.03 ng mL^−1^	[155]

Abbreviations: 2-ABA: 2-aminobenzyl amine; AFB_1_: Aflatoxin B_1_; AgNPs: Silver nanoparticles; ALVs-J: Avian leukosis virus subgroup J; AuNBs: Gold nano-bundles; CFP-10: Culture filtrate protein-10; cfu: Colony forming units; cu: Copper; CV: Cyclic voltammetry; DPV: Differential pulse voltammetry; *E. coli*: *Escherichia coli*; EIS: Electron impedance spectroscopy; FAdVs: Fowl adenovirus; Fe_3_O_4_: Ferric ferrous oxide; GCE: Glassy carbon electrode; GQDs: Graphene quantum dots; HBsAg: Hepatitis B surface antigen; HCV: Hepatitis C virus; HEV: Hepatitis E virus; ITO: Indium tin oxide; N,S–GQDs: Nitrogen- and thiol-doped graphene quantum dots; PANI: Polyaniline; PDA: Polydopamine; PS: Polymer nanosphere; pfu: Plague forming units; RNA: Ribonucleic acid; SPCE: Screen-printed carbon electrode; *S. typhimurium*: *Salmonella typhimurium*; TCID_50_: Median tissue culture infectious dose; *Y. enterocolitica*: *Yersinia enterecolitica*.
